# Initial experiences with Direct Imaging of Neuronal Activity (DIANA) in humans

**DOI:** 10.1162/imag_a_00013

**Published:** 2023-09-06

**Authors:** Shota Hodono, Reuben Rideaux, Timo van Kerkoerle, Martijn A. Cloos

**Affiliations:** Centre for Advanced Imaging, The University of Queensland, Brisbane, Australia; Queensland Brain Institute, The University of Queensland, Brisbane, Australia; School of Psychology, The University of Sydney, Camperdown, Australia; Cognitive Neuroimaging Unit, CEA, INSERM, Université Paris-Saclay, NeuroSpin Center, Gif/Yvette, France; ARC Training Centre for Innovation in Biomedical Imaging Technology (CIBIT), The University of Queensland, Brisbane, Australia

**Keywords:** functional MRI, neuronal activity, DIANA, physiological noise, artifacts

## Abstract

Functional MRI (fMRI) has been widely used to study activity patterns in the human brain. It infers neuronal activity from the associated hemodynamic response, which fundamentally limits its spatiotemporal specificity. In mice, the Direct Imaging of Neuronal Activity (DIANA) method revealed MRI signals that correlated with extracellular electric activity, showing high spatiotemporal specificity. In this work, we attempted DIANA in humans. Five experimental paradigms were tested, exploring different stimulus types (flickering noise patterns, and naturalistic images), stimulus durations (50–200 ms), and imaging resolution (2 × 2 × 5 mm^3^ and 1 × 1 × 5 mm^3^). Regions of interest (ROI) were derived from Blood Oxygen Level Dependent (BOLD) fMRI acquisitions (both EPI and FLASH based) and T1-weighted anatomical scans. In Paradigm I (*n *= 1), using flickering noise patterns, signals were detected that resembled possible functional activity from a small ROI. However, changes in stimulus duration did not lead to corresponding signal changes (Paradigm II; *n *= 1). Therefore, care should be taken not to mistake artifacts for neuronal activity. In Paradigm III (*n *= 3), when averaged across multiple subjects, a ~200 ms long 0.02% signal increase was observed ~100 ms after the stimulus onset (10x smaller than the expected signal). However, white matter control ROIs showed similarly large signal fluctuations. In Paradigm IV (*n *= 3), naturalistic image stimuli were used, but did not reveal signs of a potential functional signal. To reduce partial voluming effects and improve ROI definition, in Paradigm V (*n *= 3), we acquired data with higher resolution (1 × 1 × 5 mm^3^) using naturalistic images. However, no sign of activation was found. It is important to note that repetitive experiments with short interstimulus intervals were found to be strenuous for the subjects, which likely impacted data quality. To obtain better data, improvements in sequence and stimulus designs are needed to maximize the DIANA signal and minimize confounds. However, without a clear understanding of DIANA’s biophysical underpinnings it is difficult to do so. Therefore, it may be more effective to first investigate DIANA signals with simultaneously recorded electrophysiological signals in more controlled settings, e.g., in anesthetized mice.

## 1 Introduction

The development of functional magnetic resonance imaging (fMRI) in the 1990s revolutionized neuroscience, offering a way to non-invasively map human brain function (Bandettini, 2007; Logothetis, 2008; Ogawa et al., 1990). Yet, fMRI’s dependence on changes in the Blood Oxygen Level Dependent (BOLD) signal as a surrogate for neuronal activity limits its spatiotemporal specificity (Kim & Ugurbil, 2003; Logothetis et al., 2001; Polimeni et al., 2010; Turner, 2002; Zhao et al., 2004). In particular, Gradient Recalled Echo (GRE)-BOLD signals are biased toward the larger drainage veins near the pial surface, far from the site of neuronal activity (Turner, 2002). Combining Ultra-High field MRI with advanced techniques such as Spin-Echo (SE)-BOLD (Han et al., 2021; Koopmans & Yacoub, 2019; Zhao et al., 2004) or Vascular Space Occupancy (VASO) (Huber et al., 2017; Yu et al., 2019) can shift BOLD sensitivity towards the capillary network, closer to the area of neuronal activity, but capillaries can also respond to the activity of neurons that are relatively far away (Chen et al., 2011).

Optical measurements of neuronally driven hemodynamics suggest the spatial specificity of fMRI has not yet reached the physiological limit (Drew et al., 2011; Hillman, 2014; Sirotin et al., 2009); however, fMRI’s biggest limitation may be its temporal specificity. In small animals, it has been demonstrated that BOLD fMRI can reveal some aspects of information flow within the cortex (Jung et al., 2021; Silva & Koretsky, 2002; Yu et al., 2014). In 2002, Silva and Koretsky implemented very high temporal resolution fMRI by swapping the phase encoding and measurement loops, and reported that BOLD fMRI can differentiate the response onset time within the cortex. Yu et al. (2014) implemented line scanning fMRI which also demonstrated that fMRI response onset coincides with neuronal inputs. In 2021, Jung et al. revealed information flow in the somatosensory network by analyzing BOLD response onset time. Although surprisingly responsive (Hodono, Polimeni, Reutens, et al., 2022; Lewis et al., 2016), the hemodynamic response function is sluggish compared to the rapid fluctuations in activity observed at the level of neurons (Friston et al., 1994). Invasive techniques in animal models have already indicated that many processes in the brain are so fast and confined to such small areas that they would not be accessible with BOLD fMRI (Siegel et al., 2015; Steinmetz et al., 2019). Animal studies contribute useful knowledge; however, in the pursuit of understanding the human brain, non-invasive neuroimaging techniques are essential. To this end, a non-invasive method that can provide measurements of neuronal activity with high spatiotemporal resolution would be a valuable tool.

Over the years, various approaches have been proposed to enable more direct observations of neuronal activation through MRI (Bihan et al., 2006; Patz et al., 2019; Roth, 2023; Stanley & Raz, 2018; Yu et al., 2014). However, each of these methods has key limitations. Diffusion-weighted fMRI (Bihan et al., 2006) is easily overshadowed by BOLD effects (Hodono, Polimeni, & Cloos, 2022; Miller et al., 2007), and specific absorption rate (SAR) and peripheral nerve stimulation considerations make measurements with sub-second temporal resolution difficult. Functional MR spectroscopy (Stanley & Raz, 2018) observes activation-related variations in metabolite concentrations. This is also subject to experimental considerations such as SAR and signal-to-noise ratio (SNR), which limit its temporal specificity to ~1 s. Elastography-based functional MRI (Patz et al., 2019) provides access to high-frequency neuronal activity, but its model-based reconstruction, relying on spatial derivatives of the signal, makes it difficult to obtain a high degree of spatial specificity.

Recently, Toi et al. (2022) further extended the limits of fMRI spatiotemporal specificity by introducing a new MRI method that aims to enable Direct Imaging of Neuronal Activity (DIANA). In their work, they employed Silva and Koretsky’s acquisition strategy (Silva & Koretsky, 2002), and showed signal changes that closely followed electrophysiological recordings and captured the thalamocortical pathway.

To resolve the mystery of the human mind, non-invasive techniques that provide high spatiotemporal specificity would be instrumental. DIANA may be able to fulfill this role if it can be translated from animals to human experiments (Kerkoerle & Cloos, 2022). Here, we describe our initial experience attempting to observe neuronal activation in humans using DIANA.

## 2 Method

### 2.1 Simulations

The DIANA method is based on a Spoiled Gradient Recalled Echo (SPGRE) sequence. Depending on the exact SPGRE sequence parameters, it can take many repetitions for the magnetization to stabilize. Full Bloch simulations were performed using different T1 values to estimate the number of dummy pulses needed to reach the steady state, assuming sequence parameters from the original DIANA paper (TR = 5 ms, flip angle (FA) = 4˚ (Toi et al., 2022)). The MATLAB (MathWorks, USA) code used in these simulations can be found at https://bitbucket.org/shotahodono/diana_spgre_sim.

### 2.2 DIANA sequence implementation and setup

Our implementation of the DIANA sequence was based on a product SPGRE sequence using 50˚ quadratic phase increments. As originally proposed by Silva and Koretsky (2002), the DIANA sequence swaps the phase and measurement loops. Combined with a synchronized repetitive functional paradigm, it becomes possible to obtain extremely high temporal resolutions (e.g., TR = 5 ms), especially when used to image a single slice. Under these conditions, each trial samples the same line in k-space *M* times once for each image in the final time series. Trials are then repeated *N* times (number of phase encoding lines), each adding one line to the time series. Thus, collectively it takes at least *M* × *N* × TR to collect one fully sampled dataset, hereafter referred to as “run.”

To ensure a stable baseline signal, an option was added to enable sufficient dummy pulses to reach the steady state, and trigger signals were added to synchronize the acquisition and functional paradigm. All DIANA experiments were performed with 2 × 2 mm^2^ or 1 × 1 mm^2^ in-plane resolution and a 5 mm slice thickness (TR = 5 ms, TE = 2.4 ms, and FA = 4˚) at 7 Tesla (Siemens Magnetom, Germany) using a 32-channel head coil (Nova Medical, USA). The exact sequence implementation including gradient amplitudes can be found in Supplementary Figure 1.

### 2.3 Phantom experiments

Phantom experiments were performed to evaluate the stability of the MRI signal. After allowing fluid motion to settle (~30 min), a custom phantom containing 50 mL centrifuge tubes with different concentrations of manganese chloride was imaged using both the SPGRE and DIANA sequences. The protocol was chosen such that both measurements produced an equal number of readouts. The SPGRE collected 1024 sequential images in 2 runs, with the first 21 measurements (~2000 TR) removed to ensure that the SPGRE signal reached the steady state. Two scans of 4 runs of DIANA measurements were collected with 700 ms trials, using 2000 dummy TRs to stabilize the signal at the start.

### 2.4 DIANA paradigms

**Fig. 1.**
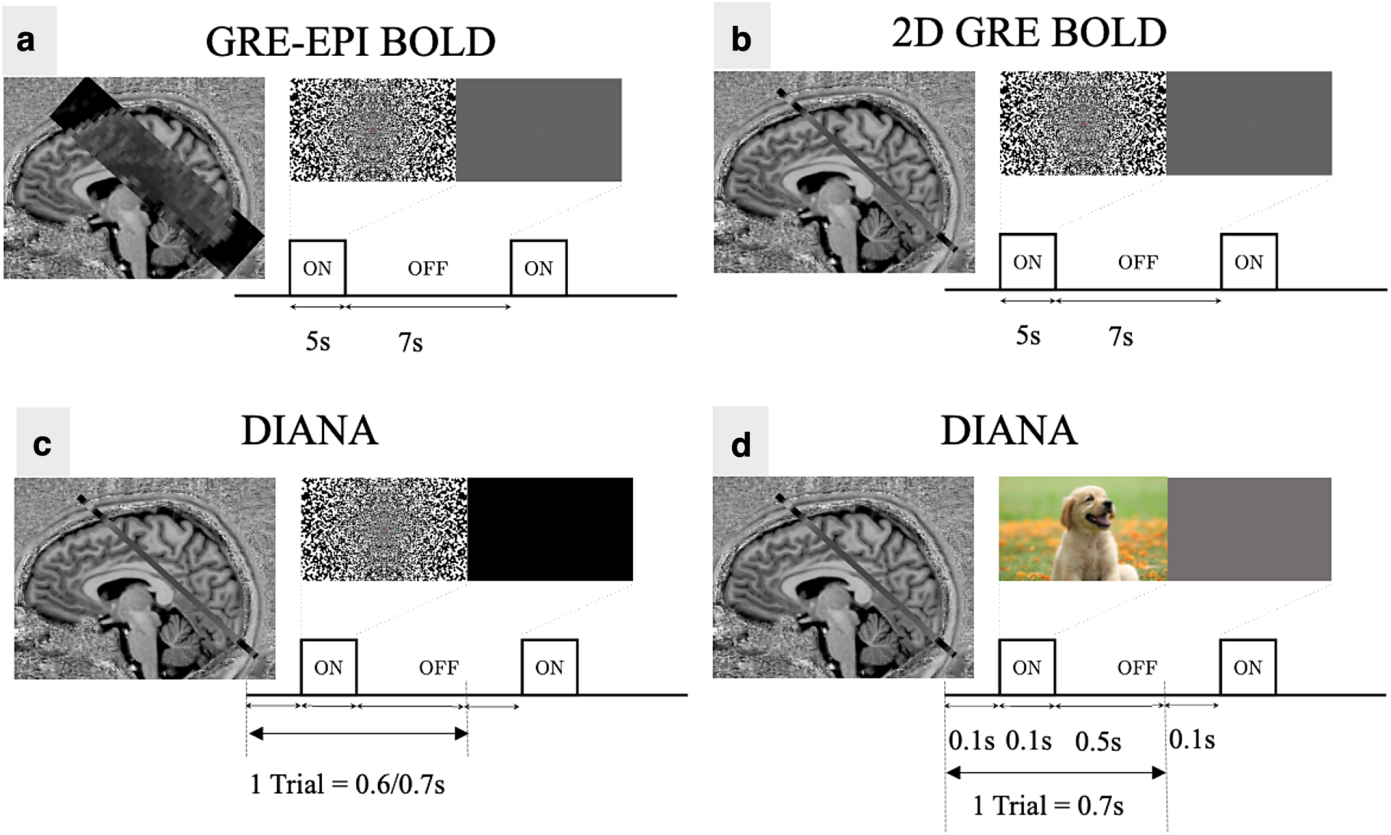
Slice placement and paradigm designs for in vivo experiments. (a) GRE-EPI BOLD for slice placement in Paradigms I, II, and III. (b) GRE BOLD for functional localization in Paradigm III. (c) DIANA acquisition in Paradigms I, II, and III. (d) DIANA acquisition in Paradigm IV.

Four different paradigms were tested, each in a different scan session (summarized in Table 1). Data for paradigms I–V were collected using a single oblique axial slice centered on the calcarine sulcus (Fig. 1).

**Table 1.** Paradigm configurations and numbers of subjects.

	Type	Stimulus duration [ms]	ISI [ms]	Number of subjects	Runs × scans	Resolution
Paradigm I	Noise	50	550	1	11 × 3	2 × 2 × 5 mm^3^
Paradigm II	Noise	200	500	1	10 × 3	2 × 2 × 5 mm^3^
Paradigm III	Noise	100	600	3	11 × 3	2 × 2 × 5 mm^3^
Paradigm IV	Naturalistic images	100	600	3	11 × 4	2 × 2 × 5 mm^3^
Paradigm V	Naturalistic images	100	600	3	11 × 4	1 × 1 × 5 mm^3^

Each trial in the DIANA paradigm consisted of a 50–200 ms visual stimulus with a 500–600 ms interstimulus interval (ISI) (Table 1). Visual stimuli were either pseudo-randomly configured noise patterns or naturalistic images that changed configuration on each trial. During ISIs, a blank (black/gray) screen was presented to minimize visual stimulation, e.g., caused by blinking. Lights in the scanner room were dimmed to reduce contrast between ambient light and closed eyes. In total, 3 scans of 10 runs were collected (1056 trials, 50 min of DIANA scan time per subject) for Paradigm I, 3 scans of 10 runs for Paradigm II, 3 scans of 11 runs per subject for Paradigm III, and 4 scans of 11 runs per subject for Paradigms IV and V.

Three human adult males and two females (23–40 yo) participated in the experiments, having provided written informed consent. All participants had either normal or corrected-to-normal vision and were screened for MRI contraindications prior to scanning. The study was approved by the local human research ethics committee in accordance with national guidelines.

### 2.5 In vivo slice placement

The DIANA imaging slice was identified using a BOLD-based GRE-EPI functional localizer (9 slices, TR = 1 s, TE = 20 ms, FA = 60˚, 2 × 2 × 5 mm, 360 volumes). A visual paradigm was used (5 s on, 7 s off; Fig. 1a) and quickly analyzed using the Fourier-transform (along the measurement dimension) to identify voxels that match the expected spectral function based on the convolution of the canonical hemodynamic response function with the functional paradigm (Hodono, Polimeni, Reutens, et al., 2022).

### 2.6 Functional localization and ROI selection

In Paradigms III and IV, the target slice was also imaged using a single slice SPGRE sequence (TR = 31 ms, TE = 20 ms, FA = 10˚, 2 × 2 × 5 mm, GRAPPA = 3, 360 volumes) to obtain a BOLD-based map of functional activity without geometric distortion (5 s on, 7 s off; Fig. 1b). In Paradigm V, GRE BOLD was modified to 1 × 1 × 5 mm, keeping all other parameters the same. The z-score maps were obtained through general linear modeling analysis with FSL (https://fsl.fmrib.ox.ac.uk/fsl/). Voxels with z-scores above 5 (in the general vicinity of V1) were used to identify BOLD-based regions of interest (ROI) where a DIANA signal may be expected.

Given the relatively low spatial resolution used in this study (2 × 2 × 5 mm), reasonable voxel-wise coincidence between BOLD and DIANA signals may be expected, even though the GRE BOLD signal is weighted towards the drainage veins at the surface (Polimeni et al., 2010; Turner, 2002). Nevertheless, we also collected a T1-weighted image of the target slice using a 2D adaptation of the MP2RAGE sequence (Marques et al., 2010). These images were used to manually draw anatomically informed ROI using ITK-SNAP (Yushkevich et al., 2006). Two control ROIs were drawn in white matter and gray matter areas where no activation is expected. We matched the number of voxels in the gray matter control ROI to the manually drawn anatomical ROI to equate the statistical power.

### 2.7 DIANA acquisition

In Paradigms I to IV, DIANA acquisition was performed with the following parameters: voxel size = 2 × 2 × 5 mm^3^, matrix size = 96 × 96, no parallel imaging, TR = 5 ms, TE = 2.4 ms, FA = 4˚, and readout bandwidth = 650 Hz/pixel. In Paradigm V, DIANA was acquired with a higher in-plane resolution, 1 × 1 mm^2^ with 5 mm slice thickness, matrix size = 192 × 192, TE = 2.3 ms, TR = 5 ms, FA = 4˚, and readout bandwidth = 810 Hz/pixel. A GRAPPA factor of 2 was used in Paradigm V so that the scan time per run remained the same, with the aim of maintaining a similar level of motion sensitivity. The sequence diagrams, including the actual gradient amplitudes, are shown in Supplementary Figure 1.

### 2.8 Image reconstruction & data analysis

All DIANA data were reconstructed offline with a 16-bit dynamic range (MATLAB, MathWorks, USA). After the reconstruction, 2D in-plane motion correction was performed. First, a reference image was computed by averaging one of the runs. Then, individual images were coregistered to the reference image using 2D rigid transformation (https://bitbucket.org/shotahodono/diana_coregi). The raw signal measured in each voxel for every trial was first converted to a percent signal change as a function of time, then linearly detrended and smoothed with a gaussian kernel (width = 3 time points). Percent signal change was then averaged across the ROI. Mean percent signal change and 95% confidence intervals were computed across the runs. Group means and the confidence intervals were computed over all runs from all subjects. The mean temporal SNR (tSNR) value in each ROI was calculated for all paradigms (Supplementary Table 1). The tSNR was calculated for each voxel based on the mean signal (120 time points for Paradigm I and 140 time points for Paradigms II, III, IV, and V) divided by the standard deviation.

## 3 Results and Discussion

### 3.1 Simulations

Bloch simulations indicated that 1000 to 1500 dummy pulses were needed to reach the steady state for all tissues (Supplementary Fig. 2). With a 5 ms TR, this translates to 10 s of dummy pulses. Given that it requires ~67 s to complete a single DIANA experiment with a 96 × 96 matrix and 140 time points (*N* = 96, *M* = 140), it is considerably more efficient to complete multiple DIANA measurements per scan. In our experiments, we ran 11 runs per scan (~12.5 min). Note that if a small number of *N* and *M* are used (e.g., when using a shorter ISI), the center of k-space can be reached before the magnetization settles into the steady state.

### 3.2 Phantom experiments

**Fig. 2.**
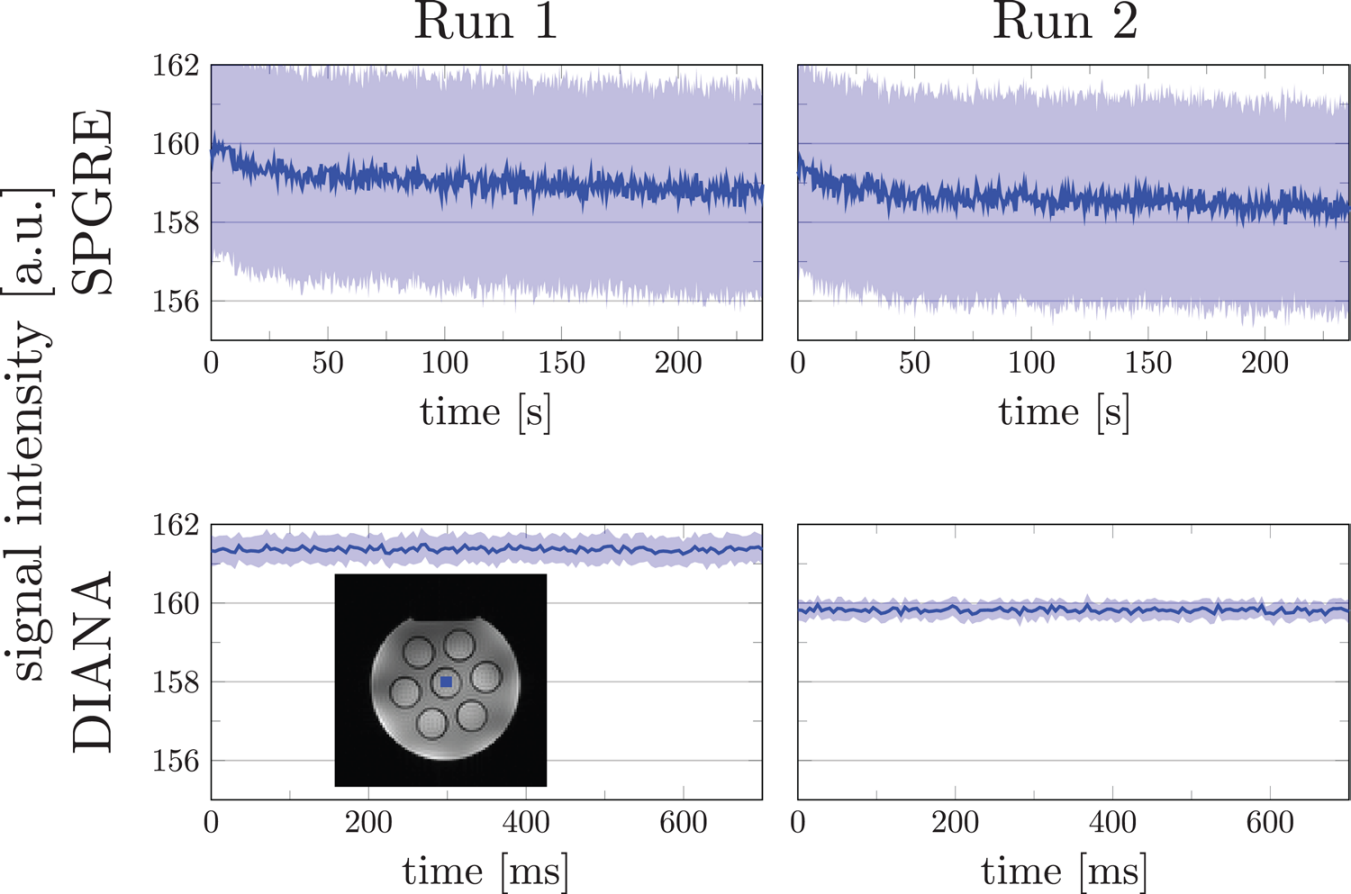
Phantom measurements showing the effect of scanner drift on traditional SPGRE and DIANA sequences. The top panels show two sequentially collected traditional SPGRE measurements, indicating a gradual drift in SPGRE. The bottom panels show two sequentially collected DIANA measurements, indicating a step change. Both measurements comprise an equal number of readouts. Blue shaded areas indicate 95% confidence intervals across voxels for SPGRE and across runs and voxels for DIANA.

The mean signal in each image is dominated by the center for k-space. The standard SPGRE sequence passes through the center of k-space once every *N* × TR, such that subsequent measurements directly reflect scanner drift (Fig. 2). Interestingly, although simulations suggested that 2000 dummy TR were adequate to reach the steady state, some samples still showed signs of a residual transient. Therefore, in the following DIANA analyses, we discarded the first run from each scan, in addition to the initial 2000 dummy TRs.

The DIANA sequence rapidly passes through the center of k-space for all time points in one trial, but then will not revisit the k-space center until *N* × *M* × TR later. Consequently, signal drift now presents itself as a step function every *N* × *M* × TR (Fig. 2). Importantly, because each trial is individually normalized based on its mean signal before averaging, these drifts are effectively eliminated from the DIANA analysis. Confidence intervals for SPGRE acquisition were calculated across voxels within ROI. For the DIANA acquisition, the mean signal over the ROI was first calculated and confidence intervals were calculated across runs. The different procedures resulted in wider confidence intervals in SPGRE acquisition.

### 3.3 Paradigms I & II

**Fig. 3.**
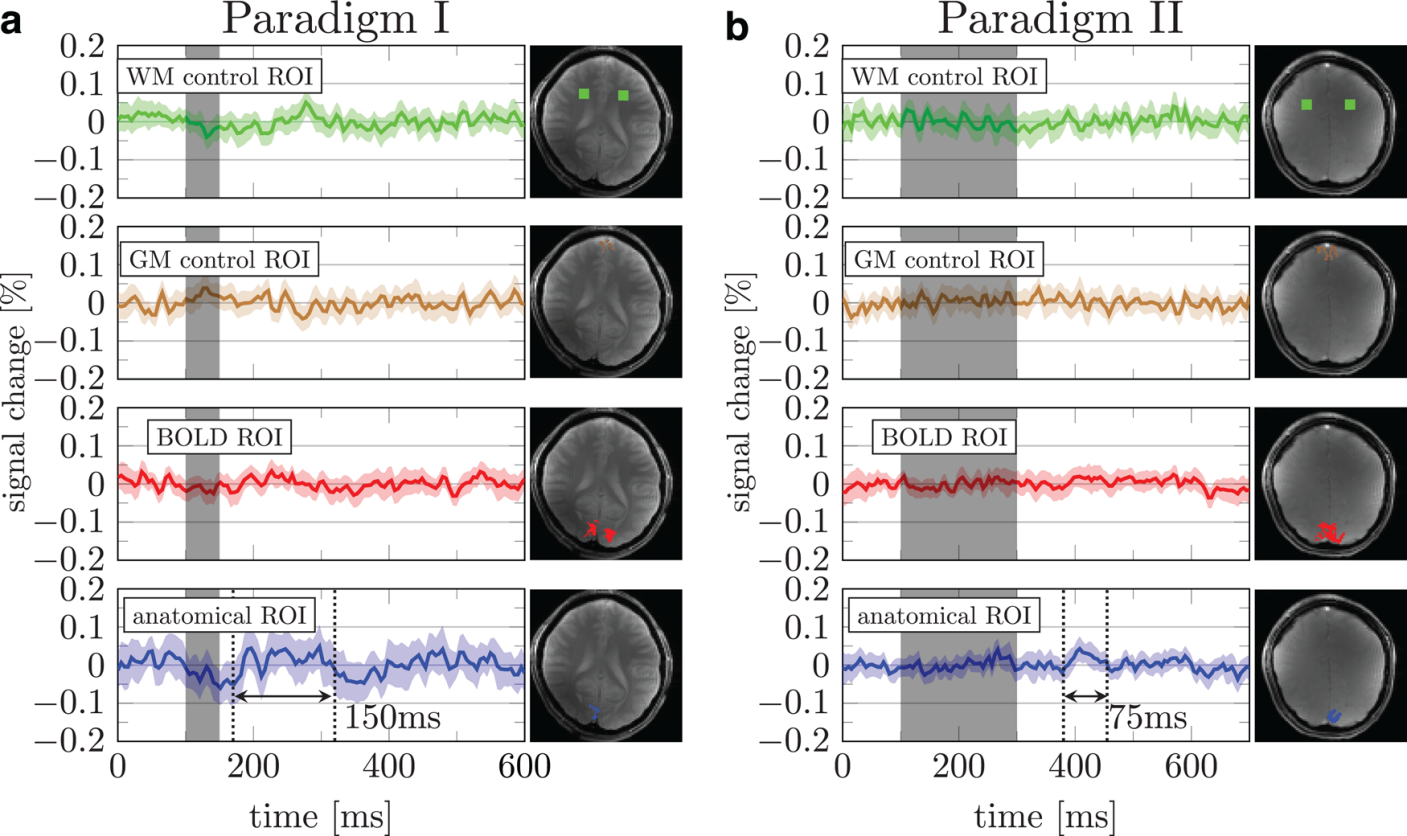
Mean response over runs using paradigms I & II. (a & b) Green, brown, red, and blue data represent signal changes from white matter control, gray matter control, BOLD based, and manually drawn anatomical ROIs, respectively. Shaded areas indicate 95% confidence interval across runs from the subject.

The DIANA signal obtained with Paradigm I showed some promise in an anatomically defined V1 ROI (Fig. 3a, blue). A 0.05% increase in signal, approximately half that observed in mice (Toi et al., 2022), followed the stimulus onset by ~75 ms. This signal increase persisted for ~150 ms. The tSNR per voxel in the ROI was >1000, and the tSNR in the ROI is expected to be increased by the square root of the number of voxels (Supplementary Table 1). Therefore, the 0.05% signal change may be detectable, albeit close to the detection limit.

Unlike the mice experiments of Toi et al., no electrophysiological recordings were available for comparison. Therefore, we devised a different test in which we increased the stimulus duration from 50 to 200 ms in Paradigm II. While some localized areas showed a peak in the time averaged signal (0.05%) after the stimulus (Fig. 3b, blue), the duration of the putative signal peak was reduced. If the change in activity was related to the stimulus, one would expect to observe a prolonged peak exceeding the duration of that observed in Paradigm I (150 ms) (Mirpour & Esteky, 2009). It should also be noted that the onset of the putative signal peak in Paradigm II is inconsistent with the temporal dynamics of the visual processing cascade. That is, the peak occurs ~300 ms after the stimulus onset, whereas responses in the early visual cortex are evoked between 50 and 100 ms after the stimulus onset (Ringach et al., 2003). It may be that the specific anatomical ROI shown in Paradigms I and II included systaltic artifacts from large vessels (Supplementary Figs. 3–5).

### 3.4 Paradigm III

The results of Paradigms I and II suggest spurious results may be found, possibly due to limited signal averaging power and poorly defined ROI. Thus, we next sought to collect more data across different participants. Further, in the following set of experiments, we also collected distortion matched SPGRE-BOLD and T1-weighted anatomical data to improve the ROI definition.

**Fig. 4.**
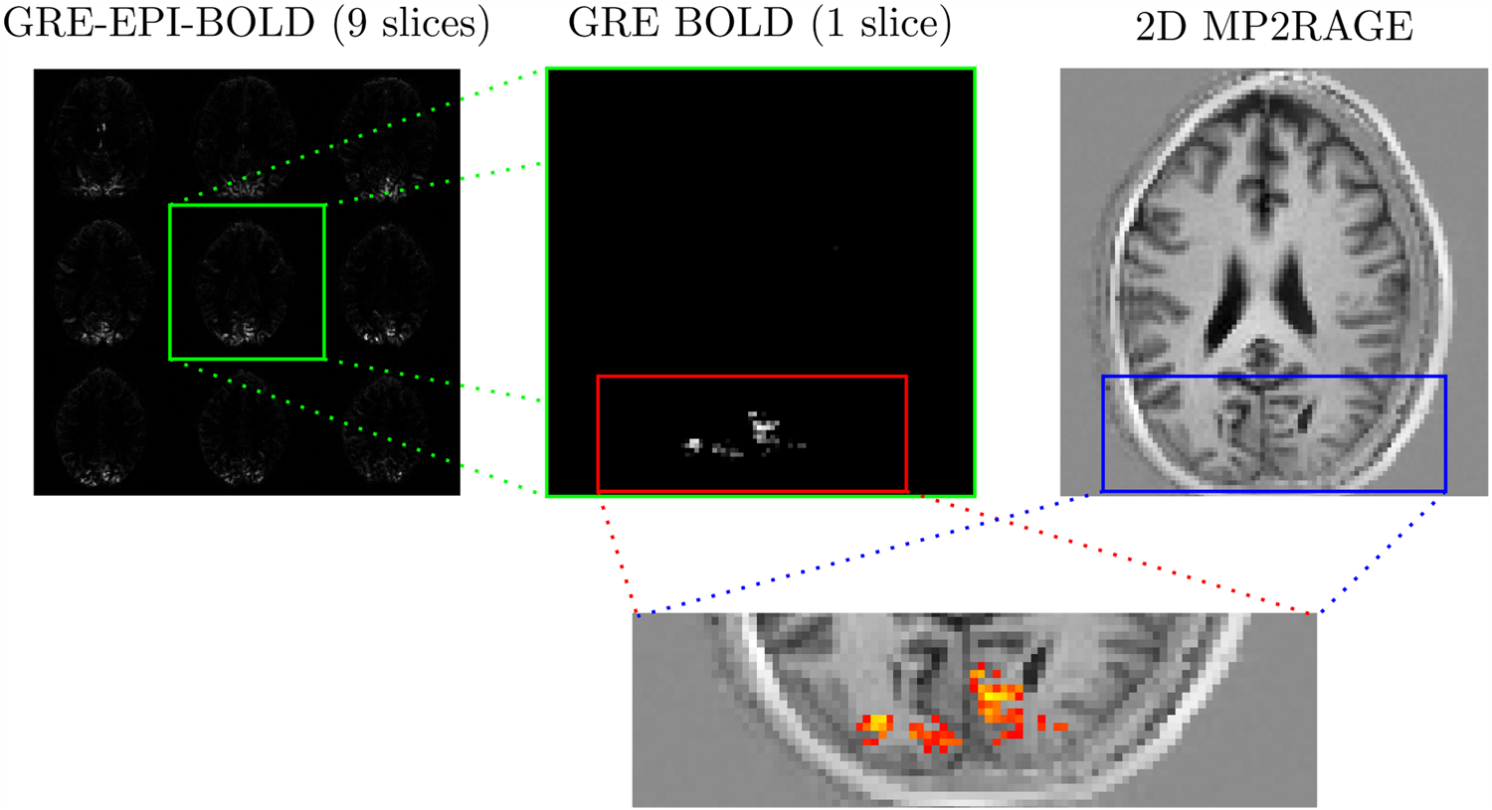
Exemplary functional and anatomical localization data from an oblique axial experiment. The left panel shows the activation estimate based on the Fourier analysis of GRE-EPI BOLD images. The middle panel shows a z-score map obtained using single slice SPGRE-BOLD. The right panel shows the T1-weighted anatomical matching the target slice. The bottom panel shows the z-score (>5) derived activation superimposed on the 2D adaptation of the MP2RAGE.

All participants showed clear stimulus-related activation across the visual cortex in the EPI-based functional localizer (Fig. 4, left). In all cases, the target slice also showed clear activation in the single slice distortion-free 2D GRE data (Fig. 4, middle), matching the expected anatomy.

**Fig. 5.**
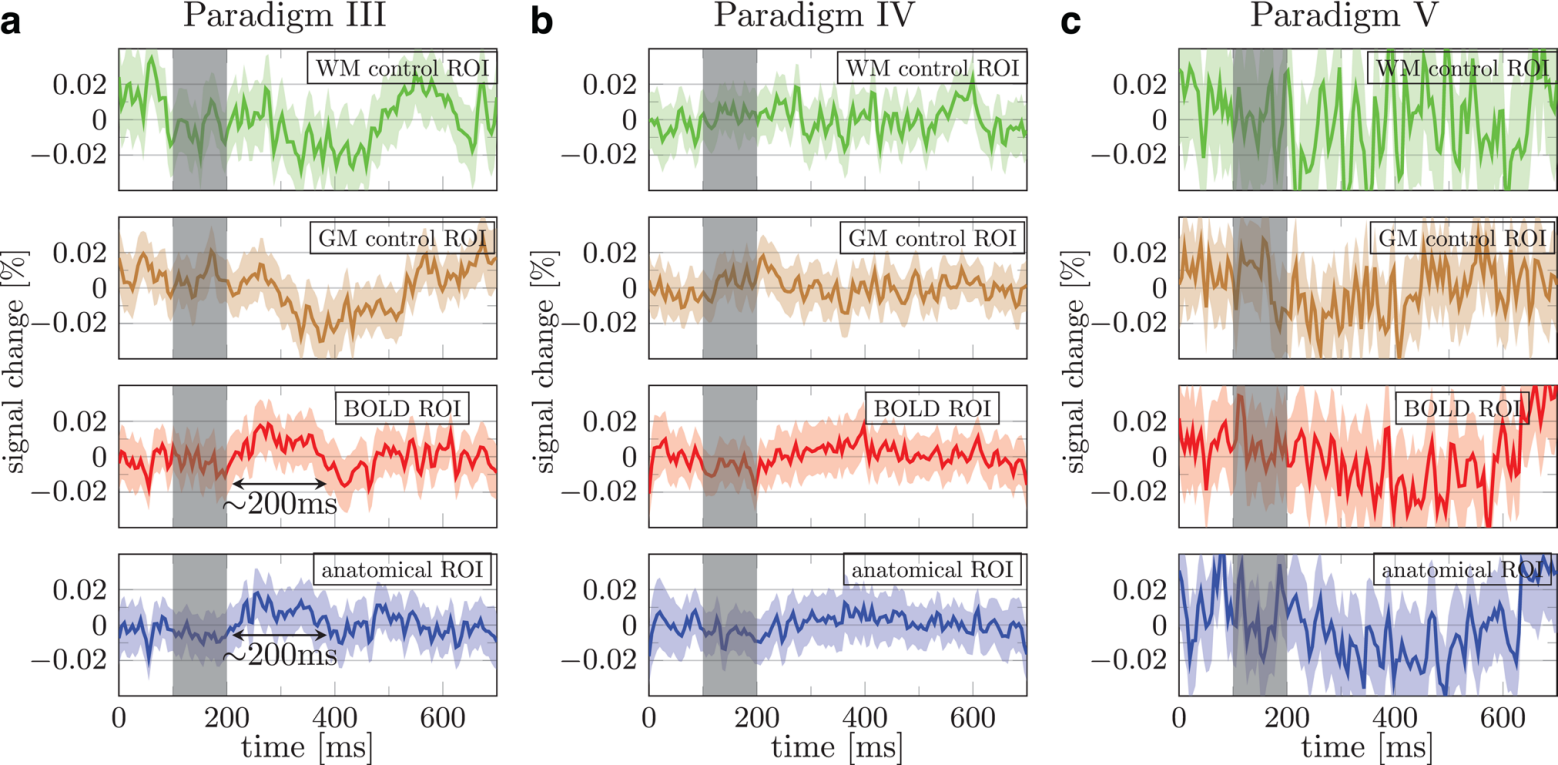
Mean signal percent change averaged across all three subjects in paradigms III (a), IV (b), and V (c). The green, brown, red, and blue data show the trial averaged signal obtained using the white matter control, gray matter control, BOLD based, and anatomical ROIs, respectively. Shaded areas show 95% confidence interval across runs from three subjects (90 runs for (a) and 120 runs for (b) and (c)). Individual subjects’ results and the ROIs are provided in Supplementary Figures 6–8.

When averaged across BOLD or anatomical ROI, trial averaged DIANA data showed no signs of activation in individual subjects (Supplementary Fig. 6, the tSNR per voxel and number of voxels in the ROIs are seen in Supplementary Table 1). When averaged across all three subjects, data from BOLD and anatomical ROIs showed a ~200 ms long 0.02% signal increase starting ~100 ms after the stimulus onset (Fig. 5a). However, the observed signal percent change was an order of magnitude smaller than what was reported in mice (Toi et al., 2022). Moreover, data from control ROI also showed signal changes exceeding 0.02%. This suggests that the signal change observed in V1 may not reflect neuronal activity.

Volunteers reported that the noise-like checkerboard stimuli with ISI ~600 ms were intense, bordering uncomfortable, motivating them to blink in anticipation. In general, blinking may be a confound in these visual paradigms using rapid stimulus presentation. A typical eye blink lasts ~100 ms, creating a visual contrast change in similar duration to the target stimulus. Although blinking could be detected using an eye tracker, data scrubbing may be difficult because DIANA inherently averages trials during image reconstruction.

### 3.5 Paradigm IV

Based on our experiences in Paradigms I–III, we surmised that tailored naturalistic images, which are more engaging and comfortable to view, may be a superior visual stimulus for the experiment.

The participants indeed reported that the naturalistic images were more comfortable, making it easier to remain attentive, allowing us to obtain 4 scans per subject (44 runs per subject). Nevertheless, the responses averaged over three subjects show no convincing evidence of a DIANA signal (Fig. 5b; data for individual subjects are provided in Supplementary Figure 7, the tSNR per voxel and number of voxels in the ROIs are seen in Supplementary Table 1).

We were unable to reproduce the positive signal change observed in Paradigm III when averaged across three subjects. It is possible that the natural stimuli produced less activation than the noise pattern used in Paradigm III. However, considering the scale of the signal, it is also possible that the increased activation found in Paradigm III was an artifact or spurious.

### 3.6 Paradigm V

In Paradigms I–IV, we employed in-plane resolution of 2 × 2 mm^2^. Despite care taken when we drew the anatomical ROIs, the relatively large voxel size is prone to partial volume effects. In Paradigm V, we used a finer in-plane resolution, 1 × 1 mm^2^, to reduce partial volume effects and improve the ROI (Supplementary Fig. 8). Although the tSNR per voxel was smaller than in Paradigms I–IV, each ROI contained a larger number of voxels, partially compensating for the reduced tSNR and per voxel (Supplementary Table 1). Nevertheless, no sign of neuronal activity was found in individual subjects (Supplementary Fig. 8) or averaged across 3 subjects (Fig. 5).

### 3.7 Recommendations for future experiments

If DIANA is possible with MRI, using the method to study human brains will require establishing an effective paradigm. In addition to visual stimuli, we also used auditory stimuli, which were more comfortable for the subject and could benefit from the high temporal specificity of the auditory system (Gazzaniga, 2000; Zanker & Harris, 2002), but ultimately failed to produce a reliable DIANA signal (data not shown). However, in the auditory experiment, the BOLD response was also notably weaker, presumably due to the auditory noise produced by the scanner itself, which may be even more problematic when using short ISIs. Finger tapping may be an effective alternative. However, care should be taken not to inadvertently induce head motion.

The expected percent change in DIANA experiments is very small (<0.5%). For individual subject analysis with reasonable scan times, such small signal changes are at the edge of the detectability. Care should be taken not to mistake small artifacts for DIANA signals (Supplementary Note). In animal imaging, the validity of such candidate signals can be tested by comparison against simultaneously recorded intracranial electrophysiological signals. However, such recordings are generally not feasible in humans. As an alternative, candidate signals can be tested by changing the experimental paradigm. In Paradigms I and II, we changed the stimulus duration. It is expected that longer stimulus block alters the response dynamics accordingly if it is neuronal. Alternatively, one could also add additional stimuli within a single trial, with the expectation that this will produce an additional signal in the trial averaged response.

In the original DIANA experiment, anesthetized mice were used. Therefore, motion was not a major issue. However, in humans, motion is likely to occur even when scanning compliant participants. Although in-plane motion can be corrected to some extent, the single slice imaging makes retrospective through-plane motion correction impossible. Real time motion correction (Lee et al., 1996; Maclaren et al., 2013; White et al., 2010) could help, but adding navigators may change the repetition time. An external tracking device may be a solution (Zaitsev et al., 2006). In addition, physiological noise contribution is also concerned (Supplementary Note); as shown in Supplementary Figure 3, there is an inflow effect. By introducing a saturation module, such artifacts may be mitigated. However, separate saturation modules lengthen the TR significantly. A clever pulse design may help reduce motion artifacts while also suppressing potential inflow effects (Hodono et al., 2023).

One other challenge is spatial resolution. In Paradigms I–IV, we employed 2 × 2 × 5 mm^3^ resolution to have sufficient SNR to detect the order of 0.1% signal change and to mitigate through-plane motion. However, such relatively large voxels may contain some amount of white matter or cerebral spinal fluid (CSF). Combined with minute motions and brain pulsations, such partial voluming effect can decrease the effective tSNR. However, high resolution acquisition will reduce the absolute SNR and require more phase encoding lines, which increases motion sensitivity. To avoid increased motion sensitivity, we employed an acceleration factor of 2, to obtain 1 × 1 × 5 mm^3^ resolution without acquiring additional phase encoding lines (Paradigm V). Even finer resolution might be feasible with higher acceleration factors. However, the SNR will drop further and g-factor noise amplification will increase (Supplementary Figure 4), which could impede the observation of DIANA responses within individual subjects and reasonable scan times.

Despite these challenges, even finer resolution might be required to resolve spatially confined neuronal signals. It can be argued that the detection of fast and transient neuronal signals necessitates not only a high temporal resolution, but also a high spatial resolution. In electrophysiology, it is commonly found that signals with high temporal frequency are coherent across smaller volumes of tissue than signals with low temporal frequencies (Bullock, 1997), and therefore also require electrodes with smaller surface areas to measure reliably (Sindhu et al., 2023; Worrell et al., 2008). In the extreme case, reliably recording single units, which are signals with a main frequency component around 1 kHz, requires an electrode with a diameter similar to that of the soma of a single neuron (Starr et al., 1973; Viswam et al., 2019). As a historical note, single unit electrophysiology, and thereby systems neuroscience (Hubel, 1982), can be said to only really have taken off with the development of a method to make electrodes that were both rigid and thin enough to allow contact sites of a few micrometers (Hubel, 1915). It could be that measuring fast transients with MRI requires a similar methodological advance.

Interestingly, a recent preprint highlights difficulties reproducing the original work in mice at very high spatiotemporal resolution at 15 Tesla (Choi et al., 2023), raising questions about the reliability and even validity of the DIANA method. An important hurdle to resolve this debate is that our current understanding of DIANA’s biophysical underpinning is still very limited. Without more detailed knowledge of the biophysical underpinning of the DIANA signal, it will be difficult to make an informed decision regarding the sequence parameterization and experimental paradigm needed to reproduce and optimize DIANA, first of all in rodents and then possibly in human imaging. To bridge this knowledge gap and in the search for non-hemodynamic fMRI signals more broadly, we believe that it may be necessary to run dedicated studies with setups that are both highly controlled and biologically representative (Morrison et al., 2023).

## Supplementary Material

Supplementary Material

## Data Availability

The data and code used for analysis are publicly available at https://osf.io/x3yab/.
