# Supplementary material for: Initial experiences with Direct Imaging of Neuronal Activity (DIANA) in humans
Source: Imaging Neurosci (Camb). 2023 Sep 6;1:imag-1-00013. doi: 10.1162/imag_a_00013 (PMC12007528; doi:10.1162/imag_a_00013)
Supplement: Supplementary Material [file imag_a_00013-supp.pdf]

Supplementary Materials

Initial experiences with Direct Imaging of Neuronal Activity (DIANA) in humans

Shota Hodono[1*], Reuben Rideaux[2,3*], Timo van Kerkoerle[4], Martijn A. Cloos[1,5]

[1]Centre for Advanced Imaging, The University of Queensland, Australia
[2]Queensland Brain Institute, The University of Queensland, Australia
[3]School of Psychology, The University of Sydney, Australia
[4]Cognitive Neuroimaging Unit, CEA, INSERM, Université Paris-Saclay, NeuroSpin center, 91191 Gif/Yvette, France
[5]ARC Training Centre for Innovation in Biomedical Imaging Technology (CIBIT), The University of Queensland, Australia
*SH and RR contributed equally to this work.

Supplementary Note 1: **Notes on physiological noise**

To study the temporal stability of the DIANA acquisition, the data collected without stimulus (1 run) was Fourier transformed along the temporal domain (Supplementary Figure 3).

The magnetization in the imaging slice is strongly saturated. Consequently, inflow effects produce strong blood signals. The Fourier transform of the DIANA timeseries clearly shows flow related artifacts aliased throughout the phase-encoding direction (Supplementary Figure 3). Following similar mechanics, cerebrospinal fluid (CSF) pulsation is also likely to produce temporally varying signal intensities.

In particular, the frequency analysis revealed artifacts at f = 1.43 Hz and f = 55.7 Hz (Supplementary Figure 4). Usually, noise related to respiration and cardiac are observed at ~0.3 Hz and ~1.0 Hz. However, our sampling rate was very high and trial window was very short (700ms). Therefore, such low frequency noise contributions might alias to other frequencies, perhaps 1.43Hz and 55.7Hz? All other frequencies except f = 0 Hz show similar noise patterns as those seen at f = 14.3 Hz. At f = 55.7 Hz, the artifacts are reduced when a finer in-plane resolution was used, which suggests that some physiological noise components can be suppressed using a smaller voxel size. However, when using parallel imaging (to reduce motion sensitivity), g-factor noise amplification comes into play too (seen at 27.2Hz). The signal intensity of these artifacts (motion, physiological, and g-factor) was three orders of magnitude smaller than the mean signal (f = 0 Hz), but can overlap with the expected DIANA signal range (~0.1 %). Therefore, care must be taken to exclude such spurious signals and future efforts could benefit from optimized imaging strategies to reduce such artifacts.

As an example, paradigms I and II showed spurious signals in select anatomical ROI (Figure 3 blue plots). For paradigm I, a general linear modeling (GLM) analysis, using the trial response average signal found in the anatomical ROI as a regressor, revealed a subset of voxels in the ROI that likely drive this spurious signal (Supplementary Figure 5). Frequency analysis revealed that high signal intensities were found in these specific voxels at 5Hz. However, both the GLM and frequency analysis also revealed additional voxels in and outside of the brain with similar properties yet clearly miss many voxels in V1. In paradigm II, GLM analysis did not reveal a distinct area driving the signal. And, Fourier analysis did not reveal a contiguous subset of voxels in the ROI at any frequency.

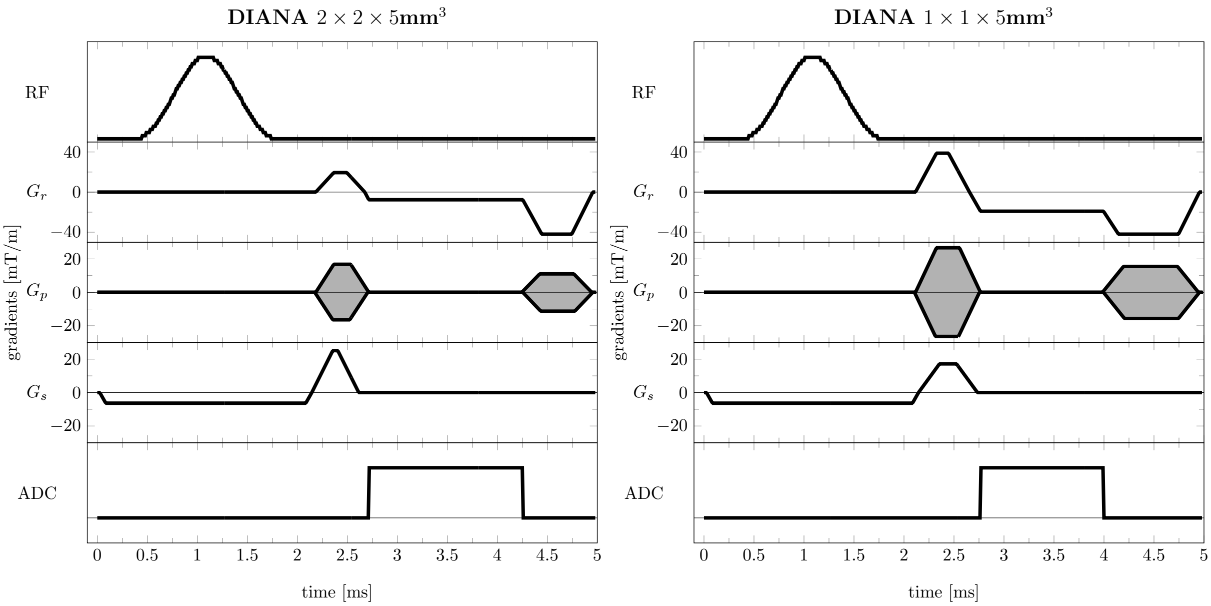

Supplementary Figure 1. **DIANA sequence diagrams showing the exact timing and gradient amplitudes used.** The amplitude in the gray shaded area depends on the phase encoding step, and the solid lines correspond to the edges of k-space.

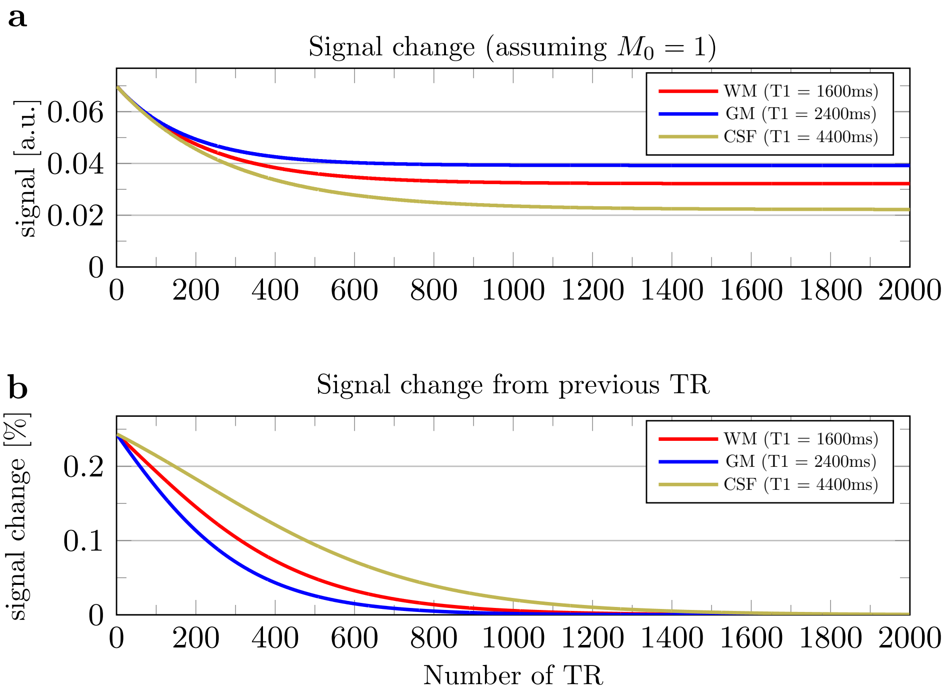

Supplementary Figure 2. **Simulations results showing the convergence of the signal towards the steady state.** The simulated SPGRE signal (assuming unit equilibrium magnetization) as a function of pulse repetition (a). The change in SPGRE signal as a function of pulse repetition (b).

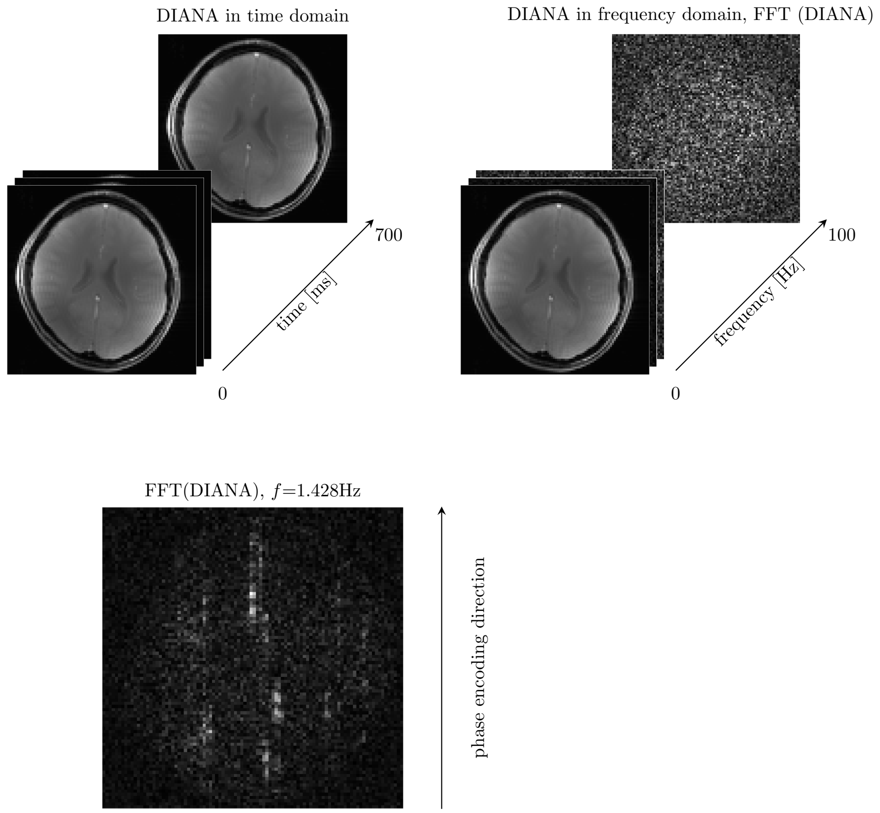

Supplementary Figure 3: **Noise analysis in DIANA acquisition**. One subject was scanned using the same experimental parameters used in paradigm III, only this time no visual stimuli were presented. The acquired images were Fourier transformed to investigate the temporal stability of the signal and look for systematic artifacts related to flow, motion, and spoiling imperfections.

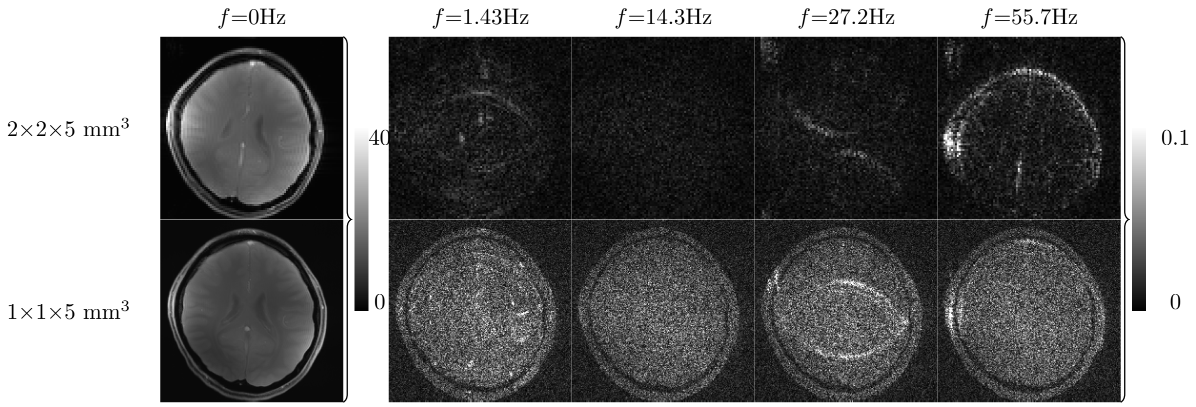

Supplementary Figure 4: **Fourier transform of DIANA images along the time dimension.** At f = 1.43 Hz and f = 55.7 Hz clear artifacts can be seen. All other frequencies except f = 0 Hz show similar noise patterns as those seen at f = 14.3 Hz. At f = 55.7 Hz, the artifacts are reduced when a finer in-plane resolution was used, which suggests that some physiological noise components can be suppressed using a smaller voxel size. However, when using parallel imaging (to reduce motion sensitivity), g-factor noise amplification comes into play too (seen at 27.2Hz). The signal intensity of these artifacts (motion, physiological, and g-factor) was three orders of magnitude smaller than the mean signal (f = 0 Hz), but can overlap with the expected DIANA signal range (~0.1 %).

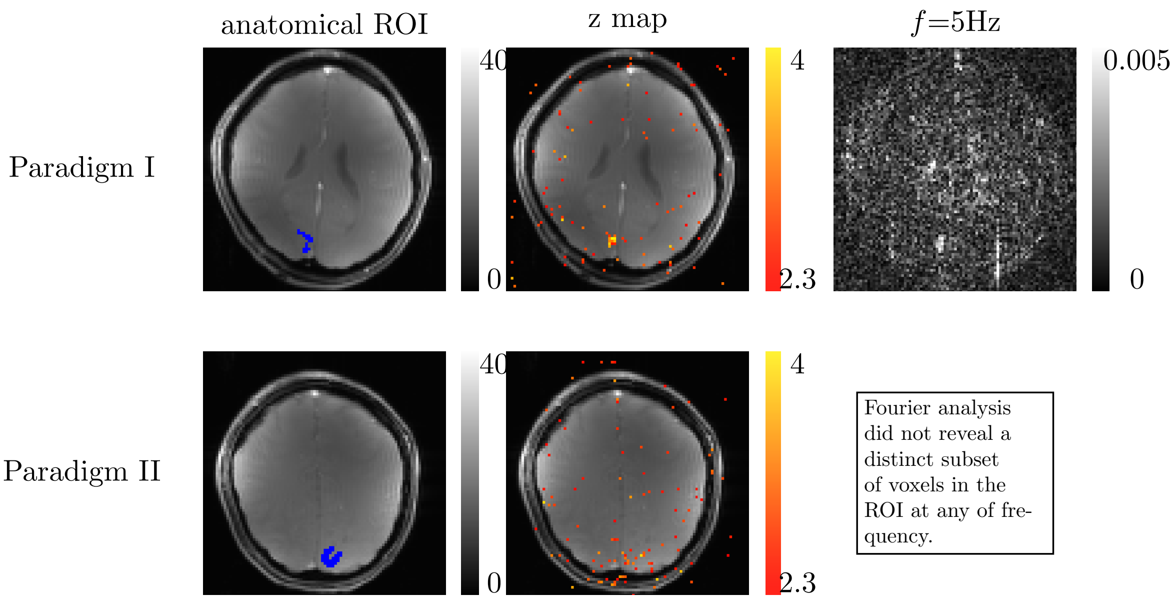

Supplementary Figure 5: **GLM based analysis for spurious signals in Paradigms I and II.** First column shows anatomical ROI localizations. Second column shows the z-score maps obtained using the signal obtained in the anatomical ROI as a regressor. Third column shows an image after Fourier transform of the time series at f=5Hz.

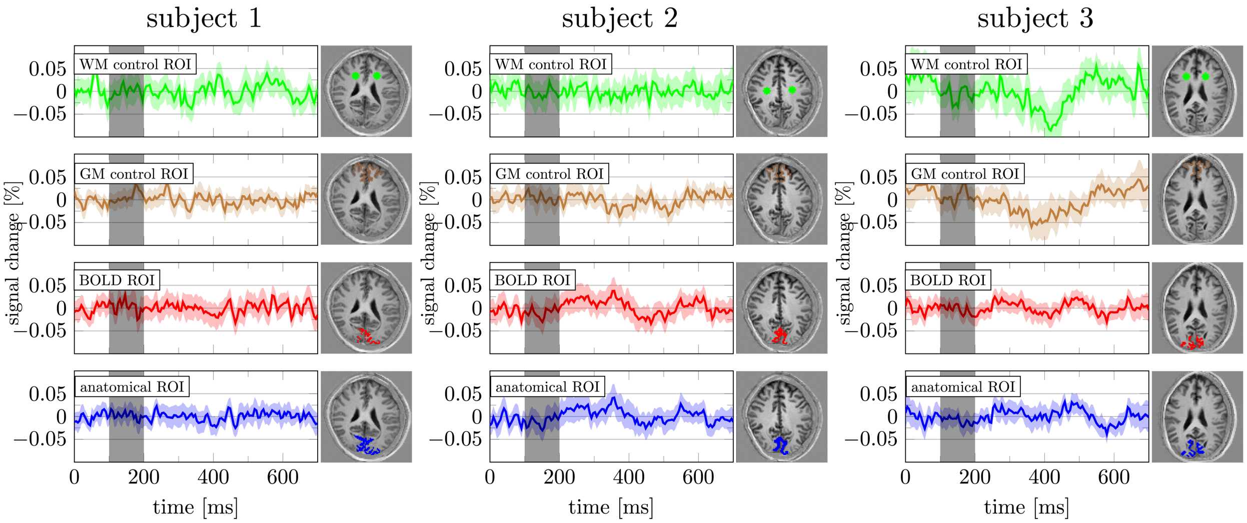

Supplementary Figure 6: **Individual subject results in paradigm III**. Three subjects (a), (b), and (c) were scanned. The green, brown, red and blue curves show the trial averaged signal obtained using the white matter control, gray matter control, BOLD, and anatomical ROIs. Shaded areas show the 95% confidence interval over runs. The black shaded areas from 100 ms to 200 ms represent the on block during which the stimuli were presented.

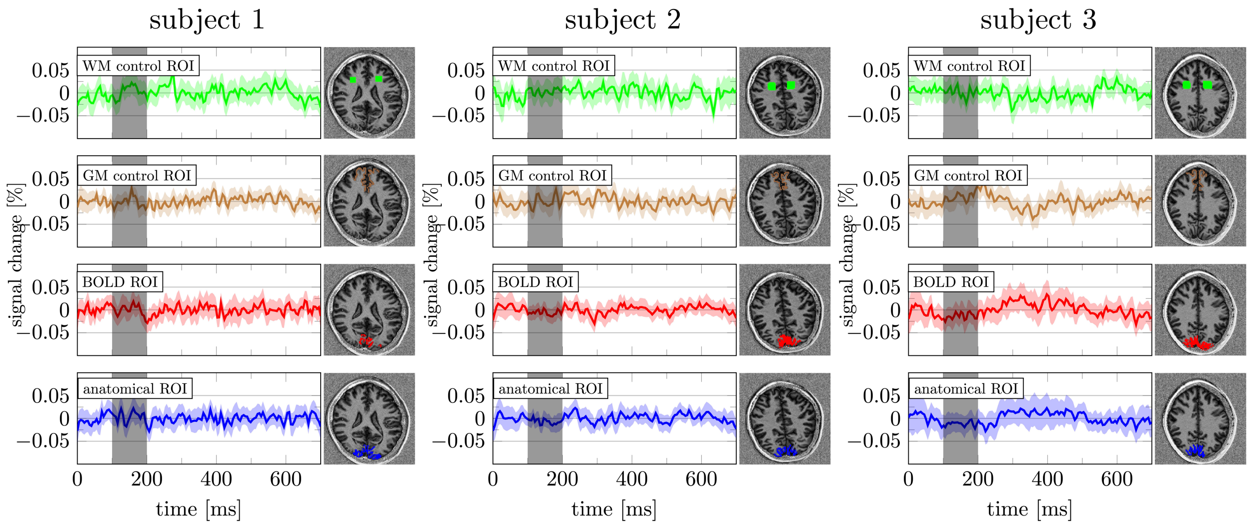

Supplementary Figure 7: **Individual subject results in paradigm IV**. Three subjects (a), (b), and (c) were scanned. The green, brown, red and blue curves show the trial averaged signal obtained using the white matter control, gray matter control, BOLD, and anatomical ROIs. Shaded areas show the 95% confidence interval over runs. The black shaded areas from 100 ms to 200 ms represent the on block during which the stimuli were presented.

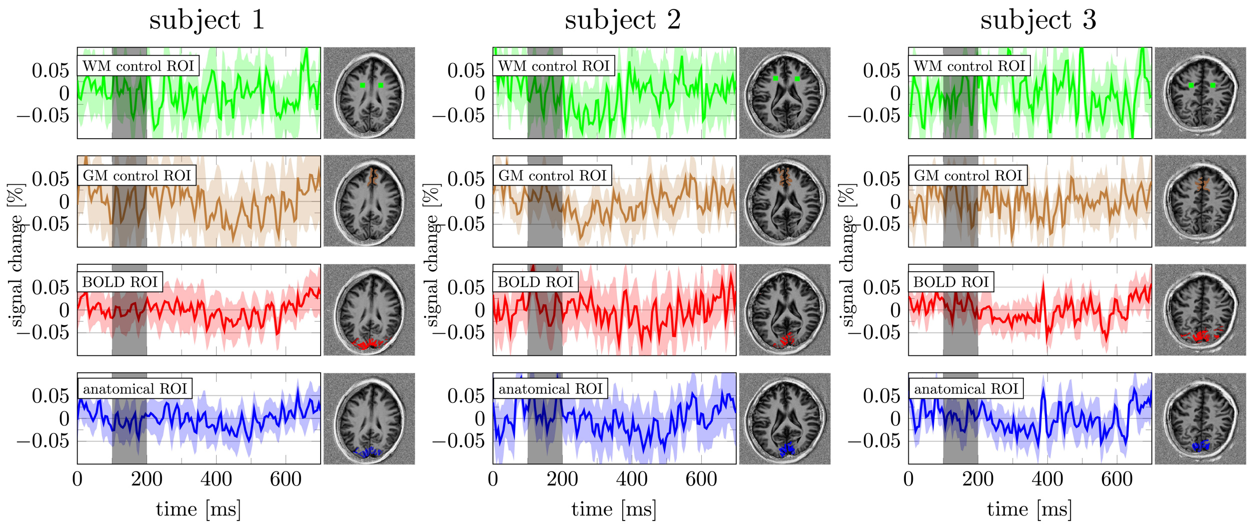

Supplementary Figure 8: **Individual subject results in paradigm V**. Three subjects (a), (b), and (c) were scanned. The green, brown, red and blue curves show the trial averaged signal obtained using the white matter control, gray matter control, BOLD, and anatomical ROIs. Shaded areas show the 95% confidence interval over runs. The black shaded areas from 100 ms to 200 ms represent the on block during which the stimuli were presented.

Supplementary Table 1: **Table of tSNR**. tSNR was calculated voxel by voxel dividing the mean signal by the standard deviation. The estimated tSNR per ROI was computed assuming that tSNR increases by a factor of square root of number of voxels.

			WM control	GM control	BOLD	anatomical
Paradigm I		tSNR / voxel	1132±147	1820±220	1369±224	1186±158
		number of voxels	72	19	65	19
		est. tSNR / ROI	9605	7933	11037	5170
Paradigm II		tSNR / voxel	1072±95	1490±250	1472±272	1531±177
		number of voxels	72	38	91	38
		est. tSNR / ROI	9096	9185	14042	9438
Paradigm III	subject 1	tSNR / voxel	1032±98	1306±256	1282±289	1261±281
		number of voxels	88	166	80	166
		est. tSNR / ROI	9681	16827	11467	16247
	subject 2	tSNR / voxel	1040±102	1413±292	1405±307	1425±342
		number of voxels	88	123	105	123
		est. tSNR / ROI	9756	15671	14397	15804
	subject 3	tSNR / voxel	1020±107	1505±276	1613±308	1537±288
		number of voxels	88	115	106	115
		est. tSNR / ROI	9568	16139	16607	16482
Paradigm IV	subject 1	tSNR / voxel	1278±108	1466±310	1412±198	1534±293
		number of voxels	78	136	67	136
		est. tSNR / ROI	11287	17096	11558	17889
	subject 2	tSNR / voxel	1147±121	1413±277	1835±373	1853±357
		number of voxels	124	101	142	101
		est. tSNR in the ROI	12772	14200	21867	18622
	subject 3	tSNR / voxel.	1425±146	1668±301	2071±632	1987±485
		number of voxels	129	105	172	105
		est. tSNR in the ROI	16185	17092	27161	20361
Paradigm V	subject 1	tSNR / voxel	283±24	400±83	469±115	478±83
		number of voxels	200	279	514	279
		est. tSNR / ROI	4002	6681	10633	7984
	subject 2	tSNR / voxel	255±22	309±50	303±48	291±47
		number of voxels	200	369	261	369
		est. tSNR in the ROI	3606	5936	4895	5590
	subject 3	tSNR / voxel.	232± 21	241±25	334±66	335±58
		number of voxels	200	322	534	322
		est. tSNR in the ROI	3281	4325	7718	6011
